# Retracted: Predictive Role of ADA in Bronchoalveolar Lavage Fluid in Making the Diagnosis of Pulmonary Tuberculosis

**DOI:** 10.1155/2014/426964

**Published:** 2014-06-05

**Authors:** Pulmonary Medicine

This article has been retracted as it is essentially identical in content with the published article “Adenosine Deaminase Activity in Bronchoalveolar Lavage Fluid in Patients with Smear-Negative Pulmonary Tuberculosis” [1] in 2008 by Tanaffos journal (Tanaffos (2008) 7(2), 45–49).
